# Supplementary material for: The Impact of Prescription Time Limits on Phosphate Administration in the Intensive Care Unit: A Before–After Quality Improvement Study
Source: Healthcare (Basel). 2024 Aug 5;12(15):1549. doi: 10.3390/healthcare12151549 (PMC11311736; doi:10.3390/healthcare12151549)
Supplement: Supplementary file 1 [file healthcare-12-01549-s001.zip › healthcare-3098164-supplementary.pdf]

**Supplementary Materials**

Phosphate replacement protocol based on serum phosphate level

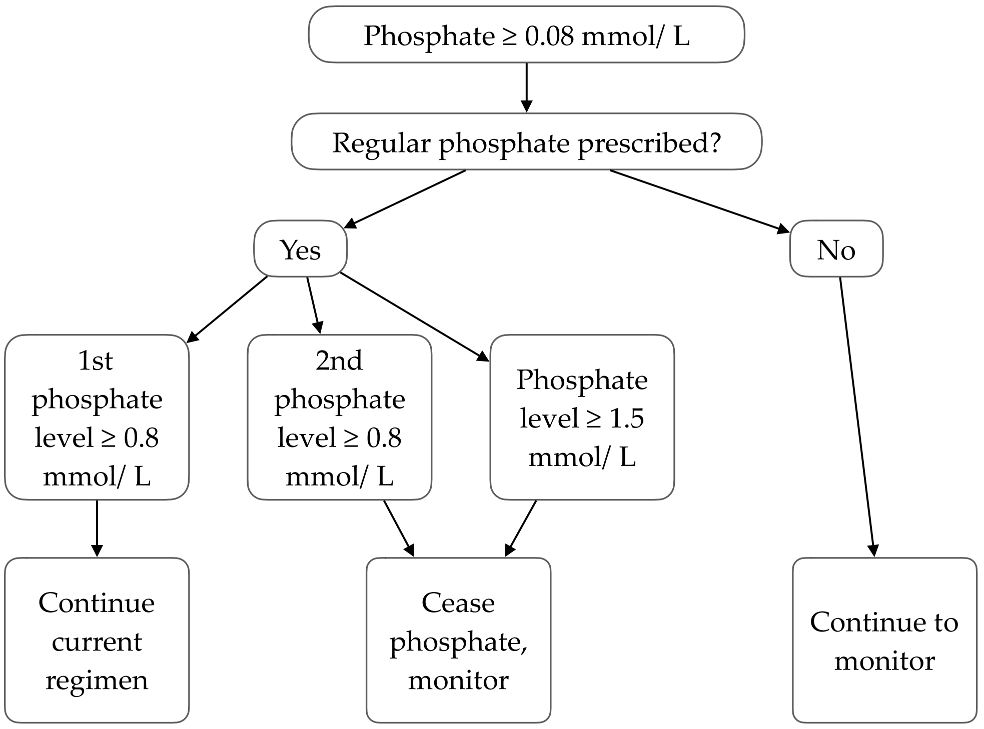


**Figure S1.** Phosphate ≥ 0.08 mmol/L.

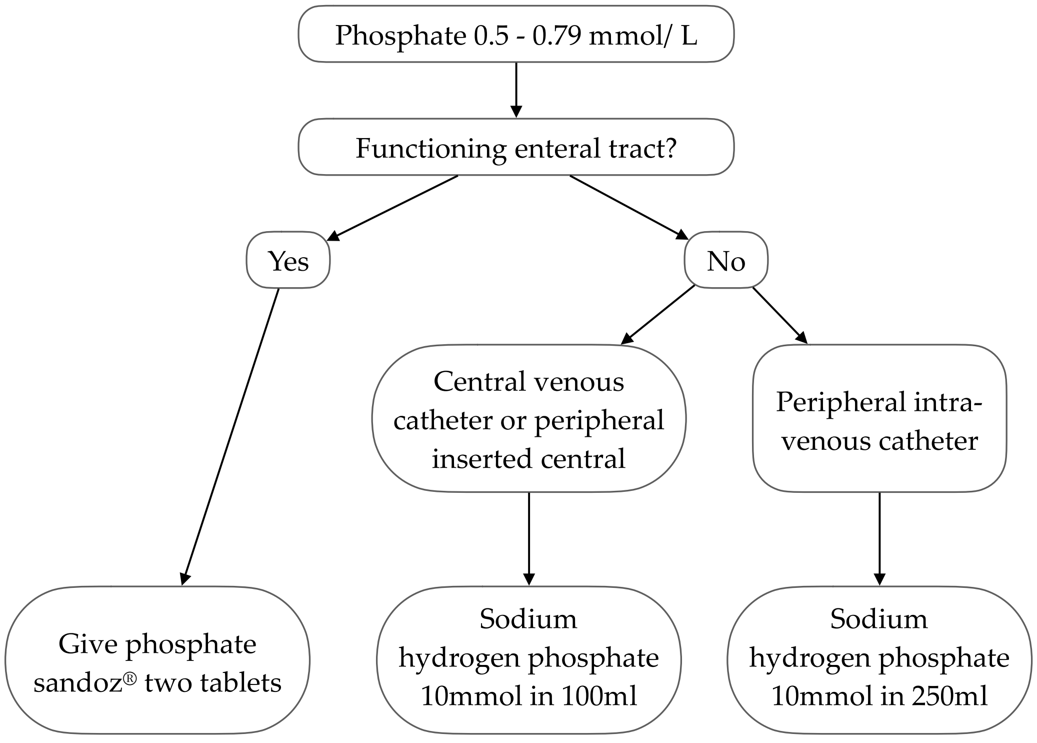


**Figure S2.** Phosphate level 0.5–0.79 mmol/L.

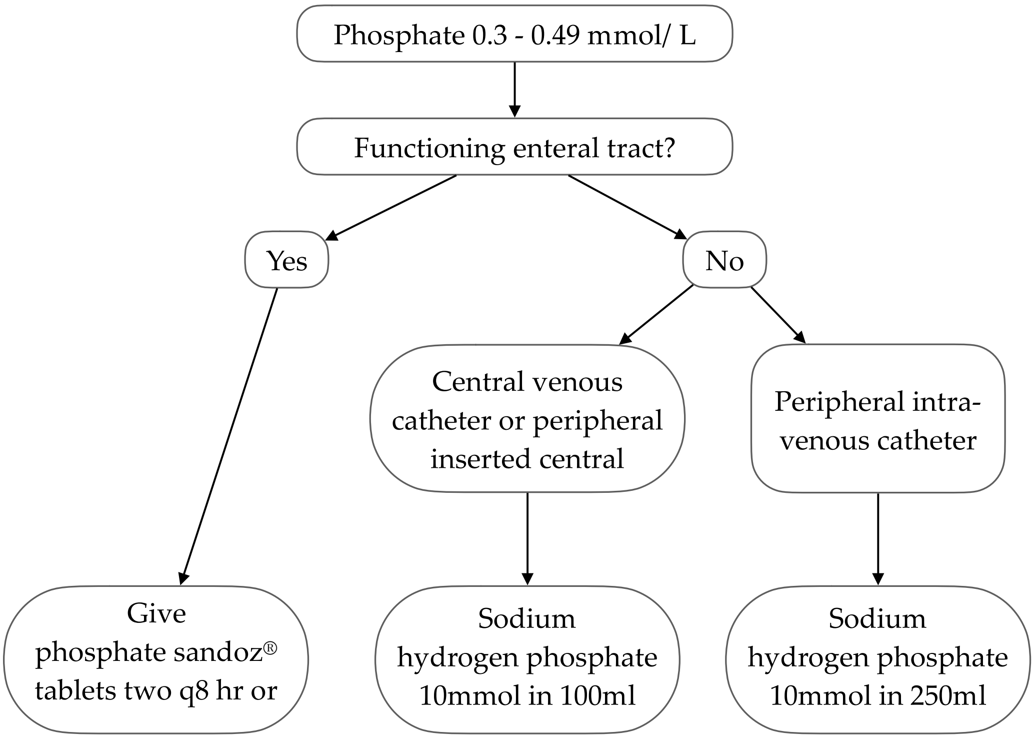


**Figure S3.** Phosphate level 0.3–0.49 mmol/L.

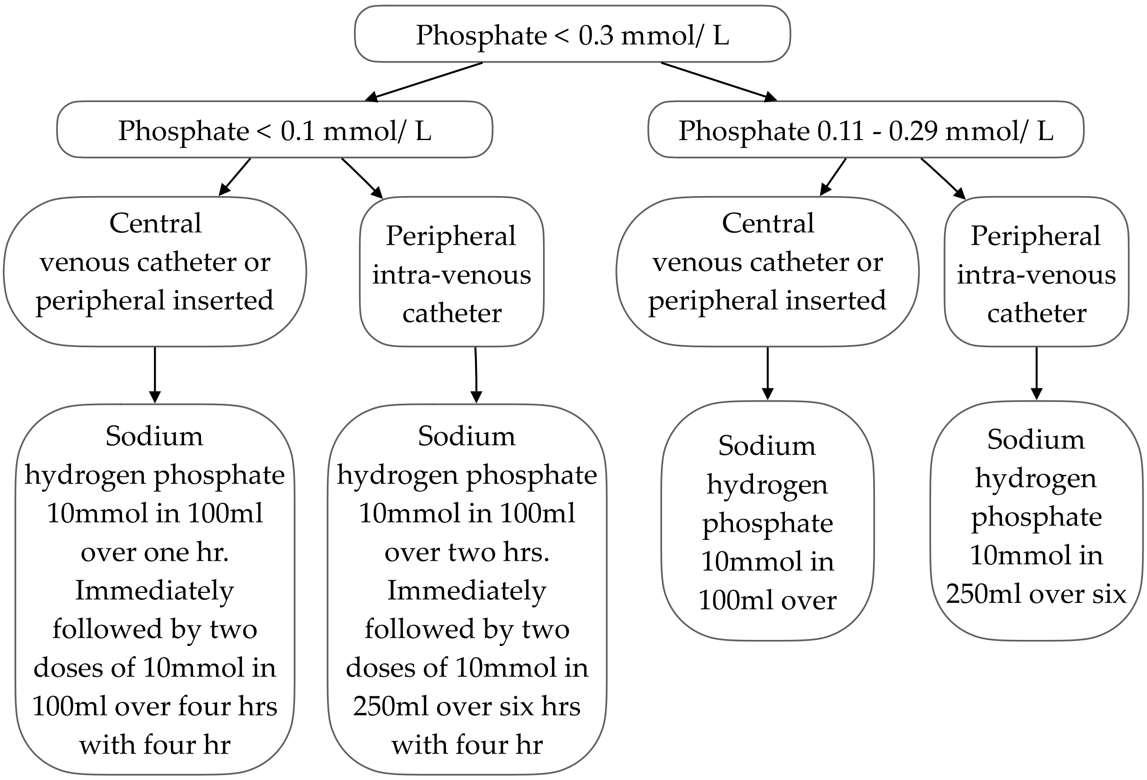


**Figure S4.** Phosphate < 0.3 mmol/L.
